# Remote Healthcare for Elderly People Using Wearables: A Review

**DOI:** 10.3390/bios12020073

**Published:** 2022-01-27

**Authors:** José Oscar Olmedo-Aguirre, Josimar Reyes-Campos, Giner Alor-Hernández, Isaac Machorro-Cano, Lisbeth Rodríguez-Mazahua, José Luis Sánchez-Cervantes

**Affiliations:** 1 Department of Electrical Engineering, CINVESTAV-IPN, Av. Instituto Politécnico Nacional 2 508, Col. San Pedro Zacatenco, Delegación Gustavo A. Madero, Mexico City C.P. 07360, Mexico; oolmedo@cinvestav.mx; 2 Tecnológico Nacional de México/I. T. Orizaba, Av. Oriente 9 852, Col. Emiliano Zapata, Orizaba C.P. 94320, Veracruz, Mexico; josi.reyescampos@gmail.com (J.R.-C.); lrodriguezm@ito-depi.edu.mx (L.R.-M.); 3 Universidad del Papaloapan, Circuito Central #200, Col. Parque Industrial, Tuxtepec C.P. 68301, Oaxaca, Mexico; imachorro@unpa.edu.mx; 4 CONACYT-Tecnológico Nacional de México/I. T. Orizaba, Av. Oriente 9 852, Col. Emiliano Zapata, Orizaba C.P. 94320, Veracruz, Mexico; jlsanchez@conacyt.mx

**Keywords:** elderly, healthcare, monitoring, sensors, wearables

## Abstract

The growth of health care spending on older adults with chronic diseases faces major concerns that require effective measures to be adopted worldwide. Among the main concerns is whether recent technological advances now offer the possibility of providing remote health care for the aging population. The benefits of suitable prevention and adequate monitoring of chronic diseases by using emerging technological paradigms such as wearable devices and the Internet of Things (IoT) can increase the detection rates of health risks to raise the quality of life for the elderly. Specifically, on the subject of remote health monitoring in older adults, a first approach is required to review devices, sensors, and wearables that serve as tools for obtaining and measuring physiological parameters in order to identify progress, limitations, and areas of opportunity in the development of health monitoring schemes. For these reasons, a review of articles on wearable devices was presented in the first instance to identify whether the selected articles addressed the needs of aged adults. Subsequently, the direct review of commercial and prototype wearable devices with the capability to read physiological parameters was presented to identify whether they are optimal or usable for health monitoring in older adults.

## 1. Introduction

Health issues are of fundamental concern for any sector of the population. However, despite the enormous efforts made to improve the detection, diagnosis, and treatment of diseases, there are areas of opportunity in those efforts carried out specifically for the elderly. Older adults are more susceptible to suffering from some type of chronic degenerative disease. Of course, there is also the risk that they will suffer more consequences by presenting non-chronic diseases considered milder for the rest of the population. It is worth mentioning that older adults represent a significant population sector since, in the last United Nations report on the aging of the world population in 2020, approximately 727 million people were aged 65 or over. Furthermore, it is expected that by the year 2050, the population of older adults will increase to approximately 1.5 billion people [1], which implies a significant economic impact on the public and personal finances of various sectors of society. Of course, the growth in economic spending on health care for older adults has a more significant impact in developing countries. In fact, in recent years, more people have been increasingly affected by chronic disorders, mainly due to the increase in the elderly population [2]. Some diseases with the highest incidence within this population sector are cardiovascular diseases, diabetes, cancer, and dementia [3].

According to the National Institute of Geriatrics of the Mexican Republic, the main reason for the loss of years of healthy life in older adults is the suffering of chronic degenerative diseases that are, in addition, the primary cause of death worldwide. In the latest statistical report published by the National Institute of Geriatrics of Mexico [4], it was estimated that the major causes of diseases in people aged 60 and over in the world correspond to:

- Cardiovascular diseases (30.3 percent);
- Cancer (15.1 percent);
- Chronic lung diseases (9.5 percent);
- Musculoskeletal diseases (7.5 percent);
- Mental disorders and diseases of the nervous system (6.6 percent).

It is imperative to monitor the health conditions of people at risk of suffering or who are already suffering from these diseases. The negative impact of these diseases is not limited to affecting the physical integrity of patients but also causes a series of secondary problems that affect the emotional and economic state of the patients themselves or even their relatives. On the contrary, there are many cases where the patient’s life is put at risk due to some side effects derived from any of the conditions. For example, there is a percentage of deaths in older adults caused by falls, which in turn can be identified as a collateral consequence of dementia or heart disease [5]. On the other hand, there is also the risk that the disease puts the patient’s life at serious risk, such as cases of premature death in the elderly associated with diabetes [6].

Although most of the ailments of older adults are caused by chronic diseases, the study results presented here are equally valid in other sectors of the population with the same ailments. Moreover, as older adults are a particularly vulnerable group with severe physical and economic limitations that drastically reduce their self-sufficiency, paying attention to this group is necessary because of their greater dependence on others such as family and friends. Though humane relationships undoubtedly promote health, there are also drawbacks such as the availability of sufficient time, knowledge, attention, and discipline necessary to follow the medical treatment that includes monitoring relevant physiological variables. For these reasons, it is crucial to determine whether the current technology of wearable devices and IoT can help effectively and reliably reduce this dependency while achieving better clinical quality in monitoring the patient’s biomedical variables. In the literature, there are already works that have reviewed some IoT and wearable devices that can monitor older adults. For example, in Wang et al. [7], three categories of wearable technology for monitoring older adults were identified and analyzed: indoor positioning, real-time sign monitoring, and activity recognition. However, the work specialized in identifying only wearable devices that allow the monitoring of intramural positioning. Additionally, Leirós-Rodríguez et al. [8] focused on the review of accelerometers that may be useful in diagnosing balance disorders in older adults, but without considering other types of diseases for the overall population healthcare monitoring. On the other hand, in Rucco et al. [9], devices for monitoring falls in older adults were included and, unlike with this work, discarding those general health monitoring devices, while in Stavropoulos et al. [10] and Tun et al. [11], a more extensive classification was made of the types of reviews focused on monitoring some chronic diseases. Additionally, in Tun et al. [11], a review was presented where the IoT technology was applied in the care of older people. Although Stavropoulos et al. [10] identified wearable devices similar to those described in this work, neither their operation nor their categorization into commercial and research prototypes was raised in their work. In addition, Tun et al. [11] focused their research not on wearable devices but on IoT technology, including devices for smart homes. Most of these reviews focused on healthcare monitoring for older adults did not include a detailed description of the devices identified, nor their grouping by diagnosed disease, nor their classification into commercial and research prototypes. The main differences between our work and other research works are three: (1) by the diagnosed diseases considered, (2) by their stage of development reached, and (3) by their FDA-approval level achieved, if any. To remark the differences, a brief comparison here is in order: (1) By the diagnosed diseases considered, Leirós-Rodríguez et al. [8] focused on alterations in balance, Rucco et al. [9] analyzed falls during static and dynamic tasks, and Wang et al. [7] focused on indoor positioning, physical activity tracking, and real-time monitoring of vital signs. Instead, this work considered cardiovascular diseases, respiratory diseases, diabetes, sleep disorders, Parkinson’s disease, alcoholism, seizures, and osteoporosis. (2) By the stage of development reached, no distinction between commercial and research prototypes was even suggested in the works of Wang et al. [7], Leirós-Rodríguez et al. [8], Rucco et al. [9], and Stavropoulos et al. [10]. The distinction is vital because only commercial wearable devices can be afforded to provide healthcare to some extent for older adults. (3) By the FDA-approval level achieved, if any, in the works presented by Wang et al. [7], Leirós-Rodríguez et al. [8], Rucco et al. [9], Stavropoulos et al. [10], and Tun et al. [11], no type of FDA approval was proposed. Likewise, others works have identified the monitoring of patients with chronic degenerative diseases [12,13,14,15,16], wearables for promoting physical activities (the deficiency of physical activity has been determined as a crucial influence in developing chronic diseases) [17,18,19], movement disorders (e.g., Parkinson’s disease, freezing of gait) [20,21,22,23,24,25,26,27,28,29,30,31], development of sensors and wearable technologies [32,33,34,35,36], wearable device use evaluations [8,10,37,38,39,40,41,42], measurement of biomedical variables and parameters [43,44,45,46,47,48,49,50], and other works have reviewed reviews related to wearables applied to healthcare [51,52,53,54]. Therefore, FDA approval for wearable devices is important because it ensures the maximum efficiency and reliability needed as the first step to providing trustworthy healthcare outside the clinic facilities.

Taking into consideration the preamble above and considering the importance of healthcare in the population sector, especially that corresponding to the elderly, the objective was to identify: (1) the physiological (medical) variables of the prevalent diseases in older adults, (2) the characteristics of the wearable devices that best fit the monitoring needs for the healthcare of older adults, and (3) the FDA assessment of wearable devices that are commercially available in the market.

## 2. Physiological Variables of Prevalent Diseases in Older Adults

**Figure 1:**
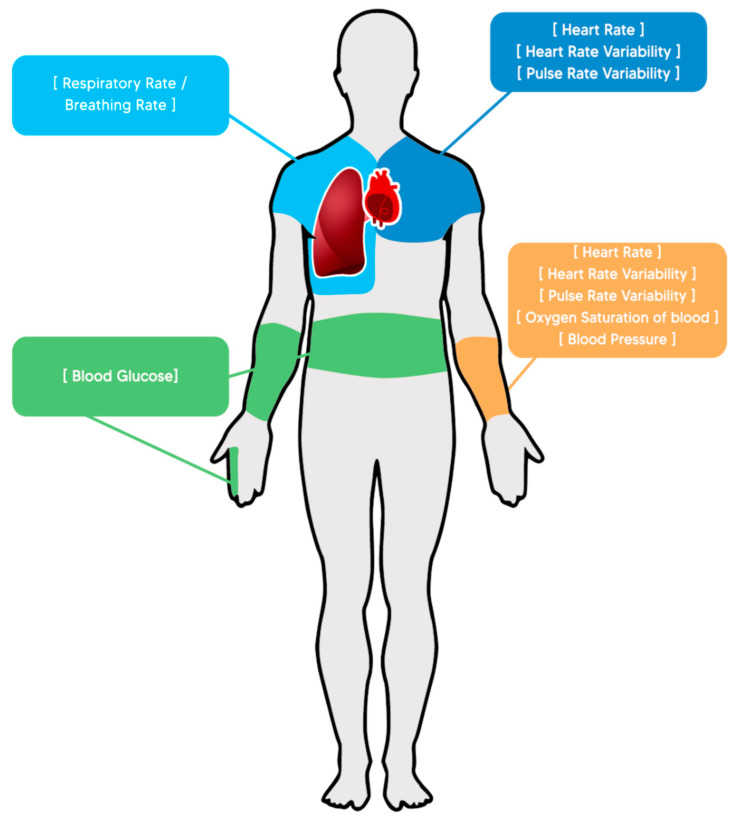
Common physiological variables and the parts of the body to which their readings are normally associated.

Once the types of diseases with the most incidents have been identified, it is possible to focus technological development efforts (in remote health monitoring issues) on devices that contain sensors capable of reading the physiological variables impacted by each type of disease. It should be noted that, due to technological limitations, it is not possible to read and monitor all the physiological variables and, therefore, all the identified diseases, still leaving areas of opportunity in terms of monitoring the health status of patients, as is the case of the state of health of cancer patients. Below, a list of the main physiological variables is presented whose monitoring allows intensive observation of patients suffering from some chronic disease. Figure 1 presents a visual representation of the same variables.

### 2.1. Heart Rate (HR)

The heartbeat frequency per minute determines heart rate. This physiological variable is widely used as an indicator of cardiac activity in different physical conditions of the human body, for example, in states of physical activity or inactivity. This variable is increasingly common because it helps monitor users who suffer from health risk conditions and is also used to measure physical performance caused by exercising [55]. HR is a particularly useful parameter in monitoring patients suffering from some cardiovascular disease within the health area. The standard technique to read HR consists of analyzing the time interval between two consecutive R wave peaks detected (QRS complex). Other data reading techniques that also consider the use of HR as a metric are ballistocardiography (BCG), phonocardiography (PCG), and impedance cardiography (ICG) [15].

### 2.2. Heart Rate Variability (HRV)

HRV is considered a physiological parameter that corresponds to the variation in the time interval between consecutive heartbeats in milliseconds and is a widely studied variable in cardiovascular disease monitoring since it is associated with heart health. Generally, high HRV values are associated with a healthy cardiac state, and, therefore, lower probabilities of death are determined. However, HRV may be affected by the gender and age of the patients. In addition, from electrocardiogram (ECG) measurements, the HR and HRV metrics can be estimated as time series of the duration of the cardiac RR intervals [56].

### 2.3. Pulse Rate Variability (PRV)

Verified in numerous studies due to the usefulness of HRV as a diagnostic and clinical research tool, pulse cycle intervals are being used instead of RR intervals as they can be obtained from photoplethysmography (PPG), which is a technique to monitor changes in blood volume in the microvascular bed of tissue. It works from a beam of light (usually green light (520 nm)) emitted by the light source (usually diode LED), which falls on the tissue of the human body; most of the light is absorbed, while the rest is reflected. The light reflected by the tissue is captured by the photosensor (receiver), which will generate an electrical signal (voltage) depending on the blood volume variation of the patient. The quality of the measurements provided by this technique depends mainly on the light emitter and receiver locations, the characteristics of the patient’s tissues, and the quality of the amplifiers and filters. Its use represents a simplification in ambulatory HRV monitoring [57].

### 2.4. Respiratory Rate (RR)/Breathing Rate (BR)

This physiological variable can be used to detect, diagnose or monitor patients affected by chronic diseases such as anxiety, asthma, pneumonia, lung disease, congestive heart failure, drug overdose, or narcotic use. A person’s RR is the number of breaths taken per minute. Usually, in adults, an RR at rest between 12 and 20 breaths per minute is considered normal, and an RR at rest is considered abnormal if it is less than 12 or greater than 25 breaths per minute. Some of the techniques under which RR recording is possible are ECG and PPG [58]. The signals obtained by the ECG and PPG have particular characteristics, such as amplitude, period, and frequency. The changes in these characteristics are used to estimate the RR through modulation schemes, such as amplitude modulation (AM), frequency modulation (FM), and baseline wander (BW). However, the presence of each of these modulations depends on each individual. The modulations may appear and disappear over time and vary depending on factors such as pre-existing health conditions, cardiopulmonary system function, gender, age, and body position. For diagnostic and monitoring purposes to provide the relevant information on the patient’s heart health, the most accepted frequency range is from 0 (direct current component, DC) to 250 Hz. However, some studies argue that it may be as high as 700 Hz [59].

### 2.5. Oxygen Saturation of the Blood (SpO2)

SpO2 refers to the amount of oxygen that is saturated in hemoglobin. Healthy adults’ good oxygen saturation value is 100%, but this can vary around 5% without considering a health risk. Some conditions that affect oxygen saturation in the blood vary from circulation problems and heart problems to respiratory problems, anemia, and congenital disabilities. One of the most common ways to measure oxygen saturation is by pulse oximetry. The readings of pulse oximetry reflect the percentage of oxygen present in the blood. Oximetry tests generally use a sensor to read the wavelengths of light reflected from the blood [60]. Pulse oximeters emit two wavelengths of light, red (660 nm) and near-infrared (near-IR) (940 nm), from a pair of small light-emitting diodes (LED) located in one arm of the finger probe. Pulse oximetry functioning relies on O2Hb and HHb differentially absorbing red and near-infrared (IR) light. It is fortuitous that O2Hb and HHb have significant differences in absorption at red and near-IR light, because these two wavelengths penetrate tissues well enough, whereas blue, green, yellow, and far-IR light are significantly absorbed by non-vascular tissues and water [61,62]. The algorithm for calculating SpO2 is based on using the Beer–Lambert law, which is the basis for estimating the percentage relationship of SpO2 between the oxygenated component of hemoglobin and total hemoglobin (made up of oxyhemoglobin and deoxyhemoglobin). The tissue is irradiated at two wavelengths (red and near-IR), and the percentage ratio of SpO2 is calculated from the absorption and reflection of the irradiated light. The calculations can be performed in the time and frequency domain. In the time domain, the changes in amplitude are analyzed to determine the SpO2. However, they are usually accompanied by random noise that causes erroneous estimates. An improvement in the SpO2 calculations can be obtained in the frequency domain, where the amplitudes of the relevant SpO2 signals stand out from the amplitudes of the random noise. This technique obtains better SpO2 estimate readings from the patient [63].

### 2.6. Blood Pressure (BP)

Blood pressure is related to the force of blood exerted against the walls of the arteries. Two numbers describe BP, as in 120/80 mm of mercury (mm Hg), where the first number, called systolic pressure, measures the pressure in the arteries when the heart beats and pushes blood in the body, whereas the second number, called diastolic pressure, measures the pressure in the arteries when the heart rests between beats. The primary disorder related to an increase in blood pressure is hypertension [64].

### 2.7. Blood Glucose (BC)

BC is a crucial physiological variable that measures the glucose concentration in the blood or plasma. Glucose is critical as a metabolic substrate for tissue energy production. Pathological conditions that affect glucose production or utilization lead to hypoglycemia. BC is considered normal if the glucose levels are between 70 and 100 mg/dl in the fasting state and less than 140 mg/dl 2 h after each meal. For this reason, continuous and effective monitoring of this variable is crucial since it must be interpreted within the clinical setting and concerning counterregulatory hormonal responses and intermediate metabolites. The leading disease related to blood glucose levels is diabetes [65].

### 2.8. Other Physiological Variables

The physiological variables analyzed in this work were restricted to only those detected by commercially available wearables or by promising research prototypes that could be introduced relatively soon into the market. Therefore, physiological variables (such as lactate, vitamins, or uric acid) that the available wearable devices cannot measure were excluded for analysis.

## 3. Methods 

This paper is a review of sensor technologies from the IoT perspective to determine if it is possible to monitor specific diseases using wearable devices and provide healthcare to older adults. For this review, due to its clarity and methodological depth, the PRISMA statement [66,67] was used only to organize and present the review more clearly.

**Inclusion and exclusion criteria.** A total of 24,615 results were obtained from all the databases. A total of 23 records were removed within the 24,615 results obtained. However, this search was refined to discard all of those published before 2010, leaving 24,592 papers. Below, we describe the inclusion and exclusion criteria.

*Inclusion criteria:* Papers related to (1) healthcare in older adults, (2) deadly, chronic, or degenerative disease, (3) commercial and non-commercial wearable devices, (4) healthcare monitoring, (5) IoT wearable devices, and (6) FDA-approved medical devices published from 2010 to 2021 were included.

*Exclusion criteria:* Papers that (1) were not written in English, (2) were not peer-reviewed, (3) were letters and reports, and (4) not primary studies were excluded.

**Information Sources.** The keywords found in the research questions could be grouped and classified according to their characteristics in the knowledge areas of healthcare and computing technology. These areas determined the specialty of the scientific digital library chosen as the source of information. For the area of healthcare, the digital scientific libraries considered were PubMed, Medline Plus, Clinical Trials, and Nice.org.uk, whereas for the area of computing technology, the digital libraries considered were IEEE Xplore, Science Direct, Hindawi, MDPI, Springer Link, Wiley Online Library, and Inderscience. These libraries were chosen because of the good results from pilot searches obtained from the federated search engine Google Scholar. From these primary sources of information, the relevant studies were extracted by submitting search queries to the corresponding search engines of each digital library. The searches were performed from January to June 2021.

**Search Strategy.** The search strategy combined keywords using Boolean-like connectives to narrow the results. The search keywords were drawn from the key concepts shaping the research questions. The search strategy derived a series of intermediate searches whose application finally led to answering the research question. Intermediate searches were ordered to find the search terms to be used in subsequent queries:

1. The main global deadly, chronic, or degenerative diseases for older people
2. The physiological variables used in diagnosed diseases
3. The sensors and biosensors that measure those physiological variables
4. The consumer wearable devices available in the market that use those sensors
5. The wearable devices that were available or not in the market
6. The FDA-approved commercial wearable devices available
7. The remote healthcare monitoring devices.

Queries 1 and 2 were applied to the medical databases. Query 1 led to the following search expression, which used adjacent search terms combined with AND and OR connectives:

‘main global disease’ AND (‘deadly disease’ OR ‘chronic disease’ OR ‘degenerative disease’) AND (‘older people’ OR ‘elderly people’ OR ‘aged population’)

The analysis of the results showed that the relevant search terms were the physiological variables referred to in the older adults’ diagnosed diseases. Query 2 included these search terms in a search expression whose execution produced new results related to physiological variables. Similarly, as the results of each of the queries listed before produced new search terms, the results were progressively expanded until reaching those that were relevant to this study.

The results at the last stage comprised those wearable devices containing sensors (biosensors) that can diagnose some major diseases in older people, including devices that can be used for remote monitoring. The included wearable devices could be either commercial or non-commercial, and in the former case, they could be either FDA-approved or not.

**Selection process.** Relevant papers were selected by title and abstract for a thorough analysis by three experts. The experts extracted the paper’s information in seven categories: brand, model, target (disease), device type, functioning, sensors (used), and FDA status. After concluding the analysis, 24241 papers were excluded. 

**Figure 2:**
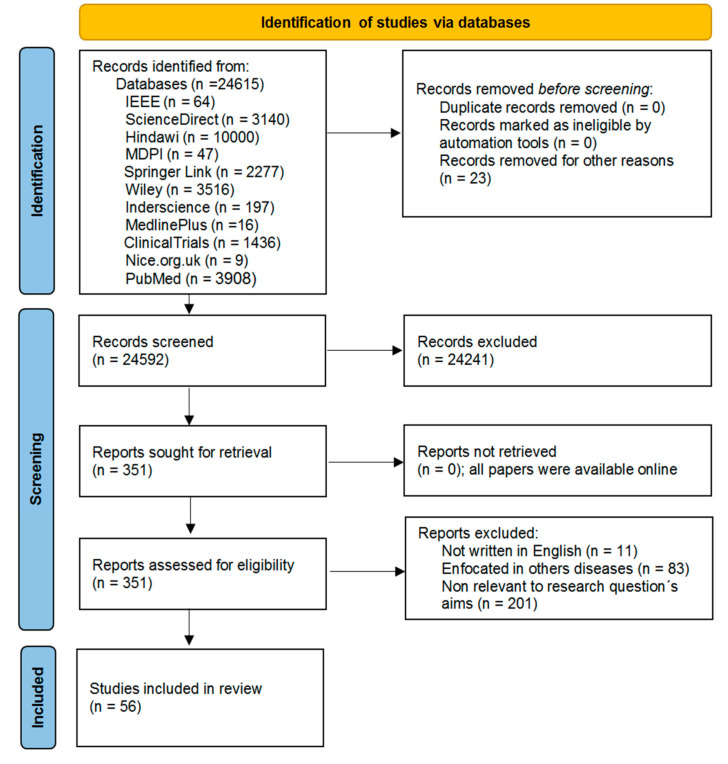
PRISMA flow diagram of the search strategy.

By reviewing the research objectives and questions of the screened papers, it was determined that 351 papers were of interest for a more detailed analysis of their content, excluding 295 papers. In addition, due to their features and detailed reviews of wearables useful for remote monitoring of health parameters in older adults, 56 articles were selected to be included in this work. In Figure 2, a PRISMA diagram represents the search strategy implemented to include the revisions presented in this document.

Finally, 56 articles were downloaded in total: IEEE (19), other sources (9), ScienceDirect (7), Nice.org.uk (7), MDPI (6), Springer Link (4), and Wiley (4).

**Data collection and analysis**. Once the selection of the papers corresponding to the primary studies concluded, the data contained in their texts were extracted and inserted in structured tables for analysis. The collected data contained information about commercial wearables and sensors for remote health monitoring. No randomized controlled selection was performed, as the number of wearables found was small (56 items). Three different researchers performed the extraction process. The characteristics of interest for the data collected on the wearable devices were: brand, model, target (disease), device type, functioning, sensors (used), and FDA status.

## 4. Results

### 4.1. Study Selection

The records identified through the information sources totaled 24,615. Figure 2 shows the composition of the results grouped by their source database. From this initial record set, 23 records were eliminated. Next, the resulting 24,592 records were screened for relevance analysis of the title and abstract, and 24,241 records were excluded after the screening. The remaining 351 records were assessed for eligibility by examining the relevance of their full-text content. The assessment excluded 295 records from eligibility for several reasons: (i) articles not written in English, (ii) articles focused in other diseases, or (iii) articles not relevant to the research question’s aims and objectives. A total of 56 records were finally included as the primary studies after applying the inclusion and exclusion criteria for eligibility.

### 4.2. Study Characteristics 

The devices identified were grouped into two large categories: commercial devices and research devices. At the time of writing this paper, among the commercial devices manufactured by technology companies, those already found on the market or with pre-sale status were included. All devices in the prototype phase, reported in scientific and research works but not manufactured yet, were classified as research devices. Below are the reviews for each category of devices, including 24 commercial wearable devices and 32 non-commercial wearable devices.

#### 4.2.1. Commercial Wearable Devices

As previously mentioned, some companies have commercialized different wearable devices whose functions allow monitoring physiological parameters that establish monitoring metrics for one or more types of disease. For this reason, a search was made for commercial devices whose characteristics allow health monitoring in older adults. It should be mentioned that most of these devices do not limit their use to older people. However, due to their capability to obtain data on physiological parameters of interest, they have been classified as suitable for monitoring diseases identified in older adults. In Table 1, a summary of the identified devices is displayed. The columns of this table correspond to the device brand and model, the diagnosed disease, the device type and its operation, a brief list of the sensors that each device uses, and the type of FDA approval. 

The table shows that there are already available in the market devices for monitoring physiological parameters needed for the care of patients with some diseases. There are general health monitoring devices that only show basic information for more relaxed health care, as is the case with smartwatches that are functional at a basic-intermediate level. It is necessary to mention that a large share of the devices developed especially for the monitoring of chronic degenerative diseases tend to meet the objective of providing readings in real-time for a better diagnosis and control of symptoms related to the identified diseases.

**Figure 3:**
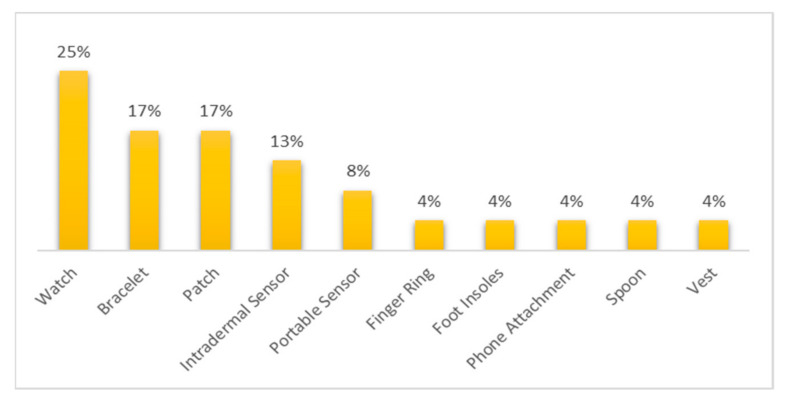
Percentage of reviewed devices classified by wearable category.

It is essential to mention that of those commercial devices that were reviewed throughout this work, it was possible to identify, taking into account their characteristics and physical mode of use, the category to which they belong. It should be noted that most of the devices reviewed belonged to the watch category (25% of devices), while the categories with the lowest numbers of devices were finger rings and foot insoles, among others (with 4% each). On the other hand, it is interesting that 13% of the devices were classified as intradermal sensors, which shows a growing interest in developing sensors that can be inserted into the human body. In addition, the portable sensor category (8%) refers to those devices that cannot be categorized as a well-known wearable type such as those discussed before. Moreover, portable sensors may fulfill their functionality in several body parts or be used alone or integrated with other sensors. Figure 3 shows in detail the percentages corresponding to the number of reviewed devices classified by category. 

**Figure 4:**
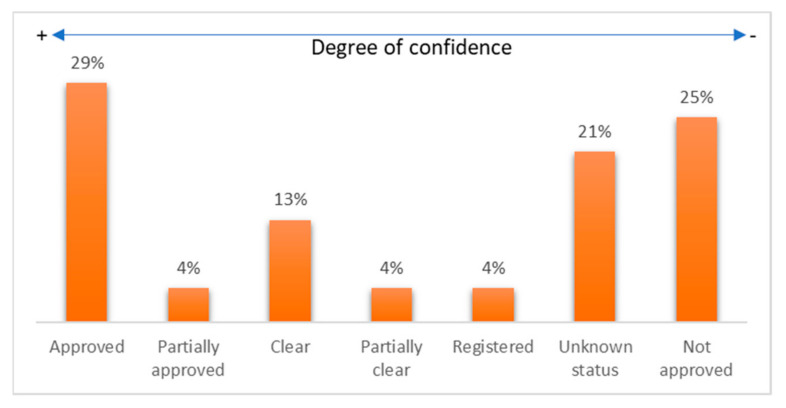
Percentage of reviewed devices classified by their FDA status.

Another type of classification is that made from the FDA status of the reviewed devices. For this classification, six statuses were considered: approved, partially approved, clear, partially clear, not approved, and registered. It is worth mentioning that many commercial devices do not have public information regarding their FDA status. It should be noted that 54% of devices have some FDA approval, while 25% are on the market without some registration or approval. On the other hand, it was not possible to find official information on the status of at least 21% of the commercial devices reviewed. Figure 4 shows the percentages corresponding to each group of devices according to their FDA status.

Likewise, it is also essential to identify the diseases for which each device is useful. A count was made of the valuable devices for each identified disease, considering whether or not they have some FDA registration. It should be mentioned that there are devices that can read physiological parameters that allow monitoring more than one type of disease condition. Therefore, some devices appear repeatedly in two or more diseases for cases that meet this condition. Table 2 presents the diseases identified for each device.

**Figure 5:**
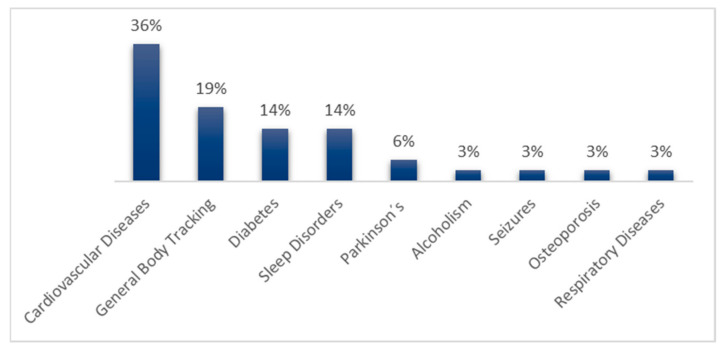
Percentage of reviewed devices useful for a particular disease.

The total number of devices for each disease. Figure 5 shows their percentages.

As shown in Figure 5, the development and commercialization of devices are focused on cardiovascular diseases. Thanks to the development of sensors and algorithms that allow reading parameters such as the oxygenation level in the blood and the heart rate, it is possible to design more and better wearable devices at a lower price for this disease category. However, other diseases are more challenging for researchers due to the difficulty of obtaining the necessary readings. An example of this situation is the case of osteoporosis, which needs constant monitoring of a patient’s bones, requiring that the relevant sensors to be implanted at the bone level. Sensor implants complicate not only device development but also device placement and maintenance.

#### 4.2.2. Non-Commercial Wearable Devices

As mentioned previously, the technologies in wearable devices that allow the monitoring of physiological parameters are in constant development. Some devices are still in development, while some others are in the prototype phase. Table 3, shown next, presents the non-commercial devices reported in research articles, whose applicability to monitoring physiological parameters makes them a viable option for follow-up and monitoring of the health status of older adults. The table is organized in columns that describe crucial aspects for health monitoring:

- Target refers to the physiological parameter that the described device can measure.
- Device Type describes the device’s category (i.e., watch and bracelet) and the year of publication.
- Functioning is a brief description of how the device works.
- Sensors Used shows the sensors found as part of the device.
- Real-Time Monitoring indicates if the device can monitor the physiological parameter in real-time.
- Elderly User Ready indicates if the device in its proposed version has the optimal characteristics and ease of use for elderly users.

It is worth mentioning that all of the reviewed research devices were developed for healthcare applications, and that more and more developers are choosing to include smaller sensors that allow the construction of more comfortable and less invasive devices. Of course, one of the primary interests in remote healthcare monitoring is to provide these devices with a greater capacity to obtain more and better physiological data and in turn communicate the data in real-time to the various health monitoring software applications to which they are connected. 

**Figure 6:**
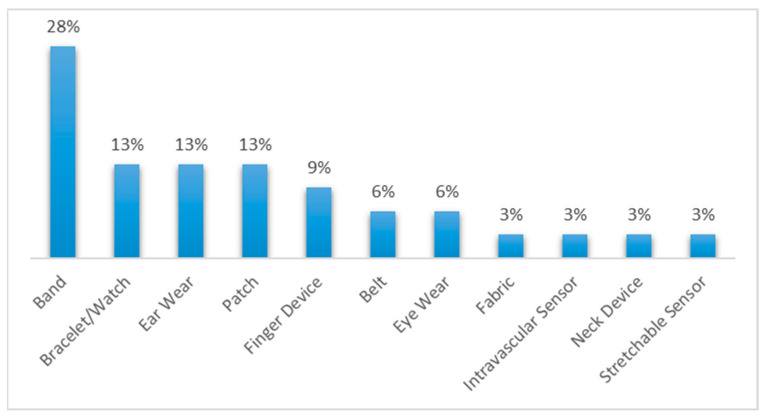
Percentages of reviewed research devices classified by device type.

Additionally, in consideration of their characteristics and modes of use, the reviewed research devices were classified into various wearable categories. Figure 6 shows the percentages of devices assigned to each category.

In turn, in Table 4, the percentages of reviewed devices and sensors that could carry out real-time monitoring were identified. In this work, real-time monitoring devices were identified as those that could transmit data on the body’s physiological variables to external devices for processing when they read the data.

Finally, Table 5 lists the number and percentage of devices that could read some of the most important physiological parameters. Some of the commercial devices were able to measure multiple parameters.

It is worth mentioning that some relevant points were identified as a subject for discussion, mainly the fact that among the devices reviewed in this work, a trend of technological development in commercial devices focused on types of cardiovascular diseases was noted, possibly derived from the current state of sensors available for the measurement of physiological parameters. For this reason, it was easier to measure parameters related to heart conditions, unlike others such as osteoporosis. On the other hand, among the research devices reviewed, it was noted that the most widely used physiological parameters had to do with the measurement of glucose and the heart rate, again, possibly derived from the general state of development of biomedical sensors. This allowed us to identify a relationship between the technological advances in sensors, their target areas, and their impact on trends in the development of manufactured and research devices.

Throughout the preparation of this work, it was possible to identify remarkable data regarding remote health monitoring in older adults. First, it was possible to identify that, of the commercial devices identified as “useful” for monitoring physiological parameters related to diseases, 36% were mainly focused on cardiovascular diseases, 19% on general body tracking, 14% on diabetes, 14% on sleep disorders, 6% on Parkinson’s, and finally, 3% each on alcoholism, seizures, osteoporosis, and respiratory diseases. Moreover, it was found that glucose and blood saturation levels, heart and respiratory rates, pulse rate and heart-rate variabilities, and blood pressure are some of the most useful physiological parameters to determine the general user’s health condition.

## 5. Discussion

### 5.1. Challenges and Trends

Throughout this work, some challenges were identified due to various factors of technological development. It is important to mention that, despite the tremendous progress that has taken place today in measuring physiological variables, there are still limitations on what can be measured. We see this mainly exemplified by the current difficulties in monitoring and measuring variables concerning difficult access areas such as the bones, lungs, or even the brain. Some of these limitations derive from the size of the components used, the methods for insertion/placement of the sensors inside the human body and their subsequent extraction, and the sensor power supplies, among others. For these reasons, there are still few control and monitoring devices available for many chronic degenerative diseases such as osteoporosis, some types of cancer, and gastrointestinal diseases, among others. These represent challenges to the development and manufacture of devices in three main categories: (1) downsizing of components and sensors, (2) device power supplies, and (3) communication/data transmission methods to access device readings.

The development and conceptualization of wearable devices that fulfill health monitoring functions is a topic of high interest within the scientific community and the business setting. Their applications can range from health care areas in controlled environments to monitoring vital signs and general body conditions with business and military applications in extreme environments. All of this has led to the exploration of new materials, architectures, communication schemes, and other aspects that can change the presentation of wearables and how they work. One of the clearest examples is the development of smart fabrics that include sensors that allow measurements ranging from body temperature to levels of electrical conductivity in the skin. On the other hand, new bio-measurement variables are being explored, including sweat as a parameter to measure metabolites such as lactate and uric acid. Likewise, the advantages of developing devices with “self-repairing” characteristics that allow them to increase their service life or ensure their operation under challenging conditions are also being investigated. Finally, we cannot fail to mention the significant trend toward the development of increasingly compact devices with extended battery life. Technological advances in wearable devices related to healthcare are presented and discussed next.

### 5.2. Emerging Solutions 

Technological development never stops, and the same may be said for the development and manufacture of wearable technology. Nowadays, some trends have been identified that are gaining interest within the scientific community regarding technological development applicable to the healthcare sector. Innovative technological paradigms such as IoT and artificial intelligence are acquiring a fundamental role in the new development proposals for sensors and mobile devices.

IoT is a technological paradigm associated with developing and improving communication schemes between devices that allows connecting devices that are increasingly smaller. Of course, this has sparked interest in, rather than connecting complete wearable devices that incorporate one or more sensors, directly connecting the individual sensors to a communication scheme that allows the collection of physiological data in real-time. Future sensors must be individually capable of transmitting large amounts of data, making them available for more efficient and timely analysis. The increasing demand for sensors in many applications, including healthcare, calls for developing smaller and more efficient batteries so that these sensors can use them individually. In other words, the goal is to achieve wireless communication from each sensor so that it is not entirely necessary for the sensor to be part of a more complex device. Thus, the conceptualization of wearable would change completely. However, achieving wireless communication with such small devices involves other challenges such as energy consumption. Therefore, the development of these independent sensors also depends on constructing more powerful batteries that supply each device opportunely. In the same way, constructing smaller and more efficient chips will allow more efficient energy use [124].

On the other hand, improvements in memory capacity, processor speed and performance, and communication throughput between sensors and applications will facilitate adopting technological paradigms such as AI, which has already been gradually integrated with wearable technology for some years. However, some solutions are still being developed to address the challenge of making individual sensors smarter to ensure that they can self-adapt to the patient’s physiological conditions such that their calibration or configuration does not need human intervention [125]. 

In addition, an increasing interest in developing more and better biosensors to measure biosignals from the human body with a more “invasive” approach should also be noted. This invasive approach allows one, among other things, to perform more reliable readings of some physiological variables such as the level of glucose in the blood [126]. As already mentioned, the idea of building increasingly smaller devices opens up a vast amount of possibilities regarding the use of sensors that can be placed or inserted in parts of the human body without being uncomfortable or obstructive [127]. The development of devices at the molecular level has already begun. It can be said that the combination of all these trends will lead to the development of more efficient, comfortable, and reliable monitoring schemes.

Many technological trends have been observed in the development of healthcare devices, favoring their autonomy, interoperability, embedded intelligence, and usability. However, these features will not be equally enhanced in all devices because their evolution is closely related to their function and user interaction. For example, the ideal device in the case of glucose control would be a stand-alone device, interoperable with data visualization applications, with sufficient built-in intelligence to determine the exact amount of insulin to be administered, and requiring no intervention in its use. Nevertheless, such a device can hardly be built with currently available technology. Autonomy is compromised because it requires incorporating both the insulin reservoir and the battery, which eventually run out. Furthermore, interoperability will be limited by the communication protocols used to send medical data, which are often rapidly rendered obsolete by new needs or new technological developments. In addition, the built-in intelligence to identify glucose patterns rising to critical levels will require increased storage, processing, and energy resources. Finally, usability is fragmented by population preferences, including, for example, aspects such as device invasiveness. Invasive devices may be the least demanding of the user’s attention, but they are generally not widely accepted, and their autonomy and interoperability are difficult to achieve, as said before. In the future, each technological trend will have a different impact on the design of each wearable device depending on its function and human interaction.

### 5.3. Limitations

The presented review has several limitations:

1. No comparative studies were included regarding the efficacy and reliability of healthcare for older people using FDA-approved wearable devices of clinical equipment grade. Comparisons with FDA-approved devices would shorten the distance in providing the basic infrastructure needed to afford good quality healthcare outside clinic and hospital facilities.

2. Among non-commercial devices, experimental prototypes based on biomarkers were not included in the study. Infectious diseases and malignant neoplasms (cancer), among others, can be diagnosed by biomarker detection. However, promising biomarker-based biosensor technology is still in the experimental phase. Known as biomarkers, biological molecules found in blood or tissues signal abnormal health conditions and can be detected by immunofluorescence or standard ELISA tests. 

3. No comparative studies were considered on the quality of life provided by commercial wearables and clinical-grade scenarios. Although remote healthcare technology is now available, many usability factors remain to be studied before the technology becomes widely accepted. The lack of acceptance of advanced digital technologies by older people and the skills required for their use are significant challenges.

4. No mobile apps were analyzed for healthcare self-management based on the wearable devices presented. The acquisition, processing, and exploration of data obtained from wearables and the notification of relevant related events ultimately require an application for analysis and decision-making. Medical data processing is an important aspect that several wearable device manufacturers have addressed through a cloud storage scheme, where specialized applications can later query the data. Furthermore, literature reviews on cardiovascular diseases [128,129,130,131,132,133] and diabetes [134,135,136,137,138,139,140,141] have described apps designed for specific diseases that were not addressed here, as they were outside the scope of this research.

5. No studies on the acceptance and skills needed for the appropriate usage of the wearables were considered.

6. No updated FDA-approval information for many wearable devices was readily available online from the manufacturers.

Among the limitations, the lack of FDA-approval grade wearable devices may impede achieving high-quality remote healthcare comparable to the care achieved with corresponding clinical equipment currently available.

## 6. Conclusions

In this work, it was found that, for wearable devices for remote healthcare monitoring for older adults, each biosensor obtains an accurate measurement of the relevant biomedical variables for the timely detection of a particular disease. The most critical biomedical variables identified were glucose, heart rate, oxygen saturation of blood, blood pressure, pulse rate variability, heart rate variability, and respiratory rate. Their importance was determined by the number of wearable devices with sensors and biosensors integrated to detect the corresponding biomedical signals of those diseases. Consequently, only those wearable devices with suitable sensors coordinated by computer applications will allow the extraction of crucial medical information that can be used to adequately monitor specific diseases. Moreover, the characteristics of wearable devices that fit the monitoring needs for the healthcare of older adults were given by their type, which directly influences device usability. In the distribution of wearable devices, the five most frequently cited types in the reviewed literature were watches (25%), bracelets (17%), patches (17%), intradermal sensors (13%), and portable sensors (8%). The question then arises as to whether biosensors integrated into wearable devices can be developed to detect lethal diseases such as malignant neoplasms (various types of cancer) in time, in which case, their state of development as final products for use across broad sectors of society will need to be determined.

In addition, after carrying out the analysis proposed in this work, it should be pointed out that not all major diseases (both due to mortality and morbidity) can be remotely monitored. In general, it can be said that most chronic degenerative diseases can be remotely monitored due to the market availability of devices, including those approved by the FDA. Regarding their medical-grade precision and reliability, the degree of user acceptance of a wearable device was determined by an FDA evaluation. The distribution of wearable devices in the market with an FDA evaluation was as follows: 29% approved, 4% partially approved, 13% clear, 4% partially clear, 4% registered, 25% not approved, and 21% with unknown status. However, for deadly diseases such as cancer that are difficult to detect in their early stages, both due to their diversity and complexity, only research allows the identification of biochemical markers that reveal the presence of cancer cells. As has been the case with drugs, whose successful approval by the FDA attests to their reliability and efficacy, this prospect of trust will eventually extend to wearable devices. In other words, those devices with FDA approval will eventually gain greater market acceptance because of the high standards of quality in their development that assured their approval.

The scope of this research was limited to wearable biomedical devices that allow obtaining valuable data in the follow-up, monitoring, and management of the health status of older adults. However, devices that can measure other physiological parameters, such as hormonal parameters, were left out. Finally, the main findings in this work were as follows:

- Among the commercial devices reviewed, 25% belonged to the smartwatch category.
- Among the commercial devices, 54% had some FDA evaluation (approved, partially approved, cleared, partially cleared, or registered).
- The diagnosed diseases that an FDA-approved wearable device can monitor were cardiovascular diseases, diabetes, general body tracking, sleep disorders, and alcoholism.
- Most of the commercial devices reviewed were devoted to cardiovascular diseases and general body tracking.
- Among the non-commercial wearable devices, those in the band, bracelet/watch, ear wear, and patch category were the most used.
- The physiological parameters that non-commercial wearable devices could monitor were glucose, heart rate, oxygen saturation of blood, blood pressure, pulse rate variability, heart rate variability, and respiratory rate.

## Figures and Tables

**Table 1:** Commercial wearables and sensors for remote health monitoring.

Brand	Model	Target	Device Type	Functioning	Sensors Used	FDA Status
Abbot	Libre 2 [68]	Diabetes	Patch	Reading of blood glucose levels.	Intradermal Glucose Sensor	Approved (2020)
AliveCor^®^	KardiaMobile [69]	Cardiology	Phone attachment	Reading the heart rate by positioning the fingers on the sensors	Electrodes	Clear (2014)
Apple	Watch 6 [70]	General Purposes	Smart Watch	Reading the heart rate by positioning the fingers on the sensors.	Oximeter, Electrical Heart Rate Sensor, Optical Heart Rate Sensor, Accelerometer, Gyroscope	ECG Approved (2018)/Oximeter not Approved
BACtrack^®^	Skyn™ [71]	Alcoholism	Bracelet	Measurement of alcohol levels.	-	Not Approved
Dexcom	G5 Mobile [72]	Diabetes	Intradermal sensor	A sensor under the skin measures glucose levels. A transmitter attaches to the top of the sensor and sends the data wirelessly to a smart device.	Intradermal Glucose Sensor	Approved (2015)
Empatica	Embrace 2 [73]	Seizures	Smart Watch	Use machine learning (ML) to detect unusual patterns that are possibly associated with seizures.	EDA Sensor, Peripheral Temperature Sensor, 3-Axis accelerometer, Gyroscope	Approved (2018)
Empatica	E4 [74]	General Purposes	Bracelet	It enables researchers to record physiological signals at home or in the laboratory. After recording, they can access the data for deep analysis.	PPG Sensor, 3-axis Accelerometer, EDA Sensor (GSR Sensor), Infrared Thermopile	Not Approved
Fitbit	Versa 2™ [75]	General Purposes	Smart Watch	It monitors the heart rate, physical activity, sleep quality, oxygen saturation, and body temperature.	3-axis accelerometer, optical heart rate monitor, altimeter, ambient light sensor, relative SpO2 sensor, built-in microphone	ECG app cleared (2020)
Fitbit	Charge 4 [76]	Cardiology	Smart Watch	It monitors the heart rate, physical activity, sleep quality, oxygen saturation, and body temperature.	3-axis accelerometer, optical heart rate monitor, altimeter	Not Approved
Health Care Originals	ADAMM [77]	Asthma	Patch	It is worn discreetly under clothing. Follow-up of cough, breathing patterns, wheezing, heart rate, skin temperature, and activity level.	Acoustic, HR, temperature	-
iRhythm	Zio^®^ [78]	Cardiology	Patch	The physiological data collected for a predefined time interval is sent by mail to the provider, who generates reports for the patient and the doctor.	ECG	Clear (2021)
Medtronic	Sensor Enlite™ [79]	Diabetes	Intradermal sensor	The sensor is inserted under the skin and captures glucose readings every 5 min, which it communicates wirelessly to the MiniMed pump or its Guardian system so that glucose levels can be observed in real-time. After 6 days, it is removed, discarded, and replaced with a new sensor.	Intradermal glucose sensing electrode	Approved (2016)
Medtronic	Guardian™ Sensor 3 [80]	Diabetes	Intradermal sensor	Once inserted, it remains under the skin, capturing glucose readings every 5 min, sending them wirelessly to the MiniMed pump or its Guardian system so that glucose levels can be seen in real-time. After 6 days, it is removed, discarded, and replaced with a new sensor.	Intradermal glucose sensing electrode	Approved (2018)
Orpyx^®^	Orpyx SI [81]	Diabetic foot	Foot Insoles	Custom insoles incorporate sensors to monitor pressure, step count, hours of wear, and temperature. Provides real-time audiovisual alerts and flushing instructions when sustained high-pressure levels occur.	Pressure sensors	Registered
Oura	Oura Ring [82]	General Purposes	Finger ring	It uses a monitoring technology that collects the heart rate, heart rate variability, temperature, activity, and sleep quality from a non-invasive ring.	Body temperature sensor, optical, infrared sensors, and a 3D accelerometer and gyroscope	Not Approved
Preventice	BodyGuardian^®^ Heart [83]	Cardiology	Patch	Small wireless monitor that adheres to the chest via a disposable strip. The strip can be repositioned as needed due to its medical-grade adhesive and electrode gel and should be replaced periodically during the monitoring period. The monitor is returned to the service provider.	Accelerometer, ECG	Clear (2012)
Sentio Solutions	Feel [84]	Emotional/mental health	Bracelet	A bracelet that monitors physiological signals throughout the day and learns to recognize emotional patterns.	EDA, PPG HR, skin sensor	-
Zoll^®^	LifeVest^®^ [85]	Cardiology	Vest	It is a portable cardioverter-defibrillator used by patients at risk of sudden cardiac death (SCD). It controls dangerously fast heart rhythms by applying an electric shock to the heart. LifeVest WCD is used directly against the patient’s skin.	Temperature sensor	Approved (2018)
Xiaomi	Mi Band 5 [86]	General Purposes	Bracelet	It monitors heart rate, physical activity, sleep quality, oxygen saturation, body temperature, menstrual cycle.	ECG	Not Approved
Withings	Move ECG [87]	Cardiology	Analog watch	In 30 s, a medical-grade ECG is ready by simply pressing the side button and placing a finger on the bezel. It can record an ECG with or without a phone nearby, as the data can be stored on the watch until the next sync.	Heart rate sensor, 3-axis accelerometer, 3-axis gyroscope	Not Approved
Huawei	Band 6 [88]	General Purposes	Smart Watch	Measurement of oxygen levels in the blood through the use of LED clusters and photodiodes. Heart rate measurement. Sleep quality monitoring.	Accelerometer, three electrodes, ECG, barometric altimeter	Not Approved
Holter	Stat-On™ [89]	Parkinson’s	Portable sensor	It is a non-invasive device worn on a belt that records the user’s motor status at all times of the day.	-	-
Gyenno	Gyenno Spoon [90]	Parkinson’s	Spoon	By detecting involuntary hand movements, sensors activate internal motors that keep the spoon stable, helping the person eat normally.	Accelerometer	-
Secmotic	Muvone [91]	Osteoporosis	Portable sensor	A device that checks if the activity carried out is appropriate to help strengthen bones or how much sun is needed to assimilate adequate amounts of Vitamin D.	-	-

**Table 2:** The number of reviewed devices that can be used for a given disease.

Disease for Which It Can Be Used	FDA Devices	Non-FDA Devices	Total
Cardiovascular Diseases	6	7	13
General Body Tracking	2	5	7
Diabetes	5	0	5
Sleep Disorders	1	4	5
Parkinson’s	0	2	2
Alcoholism	1	0	1
Seizures	1	0	1
Osteoporosis	0	1	1
Respiratory Diseases	0	1	1

**Table 3:** Research wearables and sensors for health monitoring.

Target	Device Type (Year of Publication)	Functioning	Sensors Used	Real-Time Monitoring	Elderly User Ready
Glucose Monitoring	Non-invasive intravascular glucose measuring sensor (2017)	It consists of ultra-thin skin-like biosensors on a flexible biocompatible paper battery. The battery generates subcutaneous electrochemical channels (ETC) by binding to the skin; the sensors act through the penetration of hyaluronic acid into the anode channel, the refiltration of intravascular blood glucose from the vessels, and the reverse iontophoresis of glucose to the skin surface [92].	Ultrathin skin-like biosensors	No	Yes
Glucose Monitoring	Wearable-band type visible-near infrared optical biosensor (2019)	It is a highly portable blood glucose sensor with a data acquisition time window that enables long-term, non-invasive continuous blood glucose monitoring (CGM). The biosensor exploits information from the pulsatile components that continuously measure the arterial blood volume in the wrist tissue during the change in blood glucose concentration [93].	Multi-chip sensor package of SFH7060 (OSRAM Semiconductor Inc., Regensburg, Germany)	Yes	Yes
Glucose Monitoring	Contact Lens (2018)	The human eye is read using a photon microstructure with a periodicity of 1.6 µm on a selective glucose hydrogel film functionalized with phenylboronic acid [94].	A photonic structure glucose sensor	Yes	No
Glucose Monitoring	Patch (2020)	It is a non-invasive, continuous, portable system, inspired by the anatomy of the vasculature, based on electro-magnetism (EM) for glycemic measurements. The structure of the sensor mimics the vasculature anatomy. The multiple detection system, depending on the patient’s characteristics, provides personalized monitoring [95].	EM sensors	Yes	No
Glucose Monitoring	Wearable-band type (2017)	It is an autonomous and minimally invasive portable microsystem for pseudo-continuous monitoring of blood glucose. With a shape memory alloy (SMA) microactuator, the microsystem pierces a slight wound in the skin and draws a whole blood sample from the skin [96].	Shape memory alloy (SMA)-based microactuator	Pseudo	Yes
Glucose Monitoring	Patch (2017)	It is a disposable patch-type device that measures glucose levels in sweat and automatically applies metformin, thanks to a transdermal drug delivery device [97].	Extendable sensors (humidity, glucose, pH, and temperature) are integrated in a monolithic way.	Yes	Yes
Glucose Monitoring	Band (2017)	The system induces sweat with different excretion rates at periodic intervals employing wirelessly programmable iontophoresis. The induced sweat can be immediately analyzed for glucose monitoring by integrating sensor iontophoresis electrodes on the same substrate [98].	Iontophoresis and sweat sensing electrodes for detection of Na + and Cl−	Yes	yes
Glucose Monitoring	Patch and Smart Band (2018)	It is a multifunctional wearable health management system that analyzes sweat glucose levels using a disposable sweat-based glucose detector strip and a wearable smart band [99]. It also continuously monitors vital signs (i.e., heart rate, blood oxygen saturation level, and activity).	Sensors for light-based photoplethysmography, accelerometer-based activity monitoring, and sweat-based electrochemical analysis	Yes	No
HR Monitoring	Bracelet (2020)	It is an IoT-based wearable HR monitoring smart sports bracelet. IoT technology enables real-time monitoring, storage, and analysis of data transmitted to a PC or mobile phone. After data processing and analysis, abnormal data will receive an alarm in time to track the health status [100].	heart rate sensor son7015 and step acceleration sensor mma9555lr1	Yes	Yes
HR Monitoring	Belt (2018)	It is a multifunctional portable electrical impedance tomography (EIT) system based on a high-performance application-specific integrated circuit (ASIC) active electrode that can record heart rate signals and measure humidity and ambient temperature [101].	ECG, accelerometers	Yes	No
HRV	Leg belt (2017)	It is a portable ECG sensor system that captures vital patient skin data from amplified signals detected by patched electrodes. These modules are capable of collecting 6 ECG lead signals [102].	ECG, accelerometers	Yes	yes
HR Monitoring	Bracelet (2019)	It integrates an HR measurement device using an optics-based pulse sensor and a Bluetooth-based communication module. In addition, an Android-based smartphone application receives and processes the sensor data [103].	Optical based pulse sensor	Yes	Yes
HR Monitoring	Finger case (2017)	A portable heart rate monitoring system that uses photoplethysmography (PPG). Based on the detection of the cardiovascular pulse, this method presents the analysis of light variations in biological tissues [104].	Pulse sensors	Yes	Yes
HR Monitoring	Smartwatch (2018)	It is a prototype that allows monitoring of the heart rate and the intervals between beats for some subjects. This prototype was made using the Samsung Gear S3 Smartwatch, with WebSocket library, nodejs, and JavaScript [105].	Samsung Gear S3 sensors	Yes	No
HRV	Armband (2019)	The device consists of a cuff designed to fit on the upper left arm that provides 3 ECG channels based on three pairs of dry electrodes (without hydrogel) [106].	ECG	Yes	Yes
HRV	Ear wear (2019)	It is a lightweight, portable device that continuously monitors stress in daily life by measuring electrocardiograms (ECG) and EEG. The system can be easily worn by hanging it from both ears [107].	ECG	No	No
PRV	(2019)	It is a portable device that collects PRV values in real-time. The device includes an amplifier and filter for signal accuracy. An accelerometer is used to eliminate noise due to motion. This device can transmit the acquired PPG signal wirelessly with the use of Wi-Fi technology [108].	Pulse sensors	Yes	
PRV	Wristband (2017)	A small portable device worn on the wrist detects and records gestures, arm movements, and biometric information such as skin temperature and pulse rate during sports activities using an inertial measurement unit [109].	6DOF motion sensor, temperature sensor, pulse rate sensor	Yes	No
PRV	Wristband (2018)	A portable sensing device capable of continuously monitoring cardiac movements and parameters on the wrist by using impedance plethysmography (IPG) technology. The sensor’s design consistently allows getting high-resolution measurements for up to 48 h [110].	-	Yes	Yes
PRV	Wristband (2018)	A handheld cuffless integrated system utilizes a piezoresistive tunneling sensor, achieving ultra-high sensitivity to detect slight wrist artery pressure. After the read, a circuit amplifies and converts the pulse pressure-induced signal to be wirelessly transmitted to the cloud for its storage [111].	Tunneling piezoresistive sensor	Yes	No
Respiratory Rate	Fabric (2018)	It is a smart textile based on a piezoresistive sensor element for respiratory monitoring [112].	Silver-plated nylon knitted fabric	Yes	No
Respiratory Rate	Stretchable sensor (2019)	It is an easy-to-use, low-cost, stretchable, and portable RR sensor that measures respiratory volumetric changes. The sensor is manufactured using polydimethylsiloxane substrates (PDMS) and a soft lithography technique for the stretchable sensor body. An inkjet printing technology creates the conductive circuit by depositing silver nanoparticles on top of PDMS substrates that detect inductance fluctuations [113].	RR sensor	Yes	No
Respiratory Rate	Armband (2018)	Respiratory rate is estimated from a cuff ECG using a method based on variations in the slopes of the QRS and the angle of the R wave. The estimates are compared with those obtained from the respiration signal. The cuff includes a pair of dry electrodes that record the ECG and is designed for long-term monitoring. [114].	ECG	Yes	No
Oxygen saturation of blood	Finger case (2018)	The device connects to a cloud gateway to support IoT applications using an MCU node as a data processor. The data sent to the cloud can be later accessed online for detailed analysis [115].	Photodetector	Yes	No
Oxygen saturation of blood	In-ear device (2020)	It is a device entered into the ear canal for real-time oxygen saturation measurement in the blood using a photoplethysmography sensor. It consists of green (537 nm), red (660 nm), and infrared (880 nm) emitting diodes, as well as a photodiode to measure reflected light [116].	Photoplethysmography sensor	Yes	No
Oxygen saturation of blood	Patch (2018)	It is a patch-type device that uses green light emitters to calculate oxygen saturation levels in the blood [117].	Photoplethysmography sensor	Yes	No
Oxygen saturation of blood	Neck device (2021)	An integrated PPG sensor (MAX30102 by MAXIM integrated) housed in a PCB emits red light (650–670 nm) and IR (870–900 nm). Then, the PPG sensor coupled to a photodiode quantifies light absorption. A three-axis linear accelerometer (LIS2DH12 by ST Electronics) assesses activity and eliminates motion artifacts as necessary [118].	PPG sensor, accelerometers	Yes	No
Oxygen saturation of blood	Finger case (2019)	It is a portable optical biosensor system that continuously measures pulse oximetry and heart rate using a reflectance-based probe [119].	Photodetector	Yes	No
Blood pressure	Wrist-watch (2017)	It is a wristwatch blood pressure monitor to measure blood pressure by holding the watch against the sternum wall to detect micro-vibrations of the chest related to the heartbeat. As the pulse wave travels from the heart to the wrist, an optical sensor and an accelerometer in the watch allow estimating the travel time (pulse transit time (PTT) to estimate BP [120].	Optical based pulse sensor	Yes	Yes
Blood pressure	In-ear device (2019)	A device called eBP measures BP from inside the ear, minimizing interference with the user’s everyday activities while maximizing their comfort level. Three key components provide this functionality: (1) a light-based pulse sensor connected to an inflatable tube placed into the ear, (2) a digital air pump with a controller, and (3) a BP calculation algorithm [121].	Optical based pulse sensor	Yes	No
Blood pressure and HR	Ear wear (2017)	ECG and PPG-based HR and BP monitor attachable to the ear for greater usability. It is suggested to place the ECG and PPG sensors at the back of the ears with the possibility of integrating them into glasses or headphones [122].	ECG and PPG	Yes	No
Blood pressure and HR	Glasses (2017)	It is a portable device that monitors the HR at three points on the user’s head. The lens prototype incorporates optical sensors, processing, storage, and communication components. The device continuously records the flow of reflected light intensities from the bloodstream and the inertial measurements of the wearer’s head [123].	Optical based pulse sensor	Yes	Yes

**Table 4:** The number of reviewed devices with real-time monitoring capability.

Real-Time Monitoring	No. of Devices	%
Yes	29	91%
No	3	9%

**Table 5:** The number of devices capable of measuring a specific physiological parameter.

Parameter Target	No. of Devices	%
Glucose	8	24%
Heart Rate	7	21%
Oxygen Saturation of Blood	5	15%
Blood Pressure	4	12%
Pulse Rate Variability	4	12%
Heart Rate Variability	3	9%
Respiratory Rate	3	9%

## Data Availability

Not applicable.
